# Understanding Intersectional Ageism and Stigma of Dementia: Protocol for a Scoping Review

**DOI:** 10.2196/46093

**Published:** 2023-04-11

**Authors:** Juanita-Dawne Bacsu, August Kortzman, Sarah Fraser, Alison L Chasteen, Jennifer MacDonald, Megan E O'Connell

**Affiliations:** 1 School of Nursing Thompson Rivers University Kamloops, BC Canada; 2 Department of Psychology University of Saskatchewan Saskatoon, SK Canada; 3 Interdisciplinary School of Health Sciences Faculty of Health Sciences University of Ottawa Ottawa, ON Canada; 4 Department of Psychology University of Toronto Toronto, ON Canada; 5 Department of Psychology Canadian Centre for Health and Safety in Agriculture University of Saskatchewan Saskatoon, SK Canada

**Keywords:** stigma, dementia, ageism, definitions, measures, older adults, quality of life, social inclusion, health care access

## Abstract

**Background:**

Ageism and stigma reduce the quality of life of older adults living with dementia. However, there is a paucity of literature addressing the intersection and combined effects of ageism and stigma of dementia. This intersectionality, rooted in the social determinants of health (ie, social support and access to health care), compounds health disparities and is, therefore, an important area of inquiry.

**Objective:**

This scoping review protocol outlines a methodology that will be used to examine ageism and stigma confronting older adults living with dementia. The aim of this scoping review will be to identify the definitional components, indicators, and measures used to track and evaluate the impact of ageism and stigma of dementia. More specifically, this review will focus on examining the commonalities and differences in definitions and measures to develop a better understanding of intersectional ageism and stigma of dementia as well as the current state of the literature.

**Methods:**

Guided by Arksey and O’Malley’s 5-stage framework, our scoping review will be conducted by searching 6 electronic databases (PsycINFO, MEDLINE, Web of Science, CINAHL, Scopus, and Embase) and a web-based search engine (ie, Google Scholar). Reference lists of relevant journal articles will be hand-searched to identify additional articles. The results from our scoping review will be presented using the PRISMA-ScR (Preferred Reporting Items for Systematic Reviews and Meta-Analyses for Scoping Reviews) checklist.

**Results:**

This scoping review protocol was registered with the Open Science Framework on January 17, 2023. Data collection and analysis and manuscript writing will occur from March to September 2023. The target date for manuscript submission will be October 2023. Findings from our scoping review will be disseminated through various means, such as journal articles, webinars, national networks, and conference presentations.

**Conclusions:**

Our scoping review will summarize and compare the core definitions and measures used to understand ageism and stigma toward older adults with dementia. This is significant because there is limited research addressing the intersectionality of ageism and stigma of dementia. Accordingly, findings from our study may provide critical knowledge and insight to help inform future research, programs, and policies to address intersectional ageism and stigma of dementia.

**Trial Registration:**

Open Science Framework; https://osf.io/yt49k

**International Registered Report Identifier (IRRID):**

PRR1-10.2196/46093

## Introduction

### Background

Ageism and stigma reduce the quality of life of marginalized older adults, including those living with dementia. Research shows that ageism and stigma are associated with higher rates of discrimination, lifetime victimization, social exclusion, and poorer physical and mental health [1]. Older adults are often stigmatized because of stereotypes and negative beliefs associated with old age. This type of stigma related to aging is often referred to as age stigma or ageism [2]. Ageism, like other types of stigmas (eg, stigma based on disability or a health condition, such as dementia), can exist at the individual, community, and policy levels. Ageism can occur internally or externally [2,3]. For example, ageist stereotypes can be self-internalized and create barriers to accessing health care and support services, especially for older adults with dementia. Specifically, older adults living with dementia can face intersectional or combined stigma of ageist stereotypes that overlap with dementia myths. For example, misinformation about dementia is often exacerbated by ageist stereotypes and inaccurate beliefs that dementia is a normal part of the aging process [3]. However, these myths and ageist stereotypes are harmful as they can delay a timely dementia diagnosis required for accessing support services and planning for the future. Despite this knowledge, there is a lack of understanding about the intersection and combined impact of ageism and stigma of dementia [2-4]. Accordingly, this intersection of ageism and stigma of dementia may exacerbate existing health inequities grounded in the social determinants of health, such as access to health care and support services.

Ageism and stigma of dementia are complex constructs that are often embedded within deep-rooted societal issues. More specifically, different forms of stigma (eg, stigma based on age, health condition, and disability) often converge and intersect (ie, intersectionality) to create health inequities and reduce health outcomes at the population level [5-7]. Accordingly, stigma toward individuals based on their age cannot be addressed separately from stigma based on individuals’ health conditions, such as dementia.

Any intervention designed to mitigate the combined effect of ageism and stigma of dementia requires knowledge of the core definitions, indicators, and measures used to track and evaluate their outcomes. Currently, there is a paucity of literature and no scoping review examining both ageism and stigma. However, a review is necessary to provide evidence-informed research of key measures to identify, monitor, and evaluate the impact of ageism and stigma of dementia. Accordingly, our scoping review will focus on understanding the core definitions and measures used to address ageism and stigma toward older adults living with dementia.

### Stigma

In Goffman’s renowned work, “Stigma: notes on the management of spoiled identity” [8], stigma is defined as attributes or characteristics that are socially discrediting. In comparison, Corrigan and Watson [9] describe stigma in terms of stereotypes (negative beliefs), prejudice (agreement with beliefs), and discrimination (discriminatory actions or behaviors) against themselves (self-stigma) or a group of people (public stigma). Corrigan and Watson [9] further explain that prolonged exposure to stigma contributes to the internalization of self-stigma. Link and Phelan [10] suggest that stigma involves components of stereotyping, labeling, and categorization of separation between “us” and “them,” which can lead to social exclusion, rejection, and discrimination. Although several definitions of stigma exist, there remains little consensus on a shared definition of stigma [11]. Without a shared definition, it is difficult for researchers to compare, monitor, and evaluate the impact of stigma on dementia.

### Ageism

The term ageism was first coined by Robert Butler [12] to define structural stereotypes and discrimination against older adults based on perceived old age. Ageism is often described in terms of negative beliefs, discriminatory actions, and stereotypes (e., severely impaired) toward older adults held by young, middle-aged, and older adult age groups [5,13,14]. Issues of ageism transcend our societal fabric through cultural values, beliefs, and media [15]. Research shows that ageism negatively impacts the health equity and outcomes of older adults. Specifically, ageism is linked to reduced physical health, poorer mental health, and earlier mortality [16,17]. Ageism is also associated with risky behaviors that impact health outcomes, such as excessive alcohol consumption, smoking, addiction, poor diet, and a reduced quality of life [5,18].

### Rationale

Ageism and stigma toward older adults can lead to serious consequences, including depression, social isolation, feelings of shame, and social exclusion [5,19]. More specifically, research shows that older adults with disabilities are often shunned, excluded, and stripped of their social status [20]. Despite this knowledge and the fact that dementia is one of the major causes of disability, few studies examine the intersection of ageism and stigma toward older adults with dementia [5]. However, to address the intersectionality of ageism and stigma of dementia, a comprehensive understanding of the definitional components and measures is required. Accordingly, the objective of this scoping review will be to identify the existing definitions and measures used to track and evaluate the impact of ageism and stigma on older adults with dementia. More specifically, this review will explore the commonalities and differences in definitions and measures to develop a better understanding of intersectional ageism and stigma of dementia.

## Methods

### Scoping Review Framework

Guided by Arksey and O’Malley’s [21] scoping review framework, our review will include the following five steps: (1) identification of the study’s aim; (2) examination of relevant studies; (3) study selection; (4) extraction of the data; and (5) collating, summarizing, and reporting the research findings. Arksey and O’Malley [21] propose an optional consultation process (step 6) involving key stakeholders to provide input in the scoping review (eg, references, interpreting the findings, and dissemination strategies). However, due to limited time and financial resources, this step will not be included in our study.

### Step 1: Identification of the Study’s Aim

The aim of the scoping review will be to synthesize the current state of the research by identifying the definitional components, indicators, and measures used to track and evaluate the impact of ageism and stigma of dementia. More specifically, this review will examine the commonalities and differences in definitions and measures to gain a better understanding of intersectional ageism and stigma of dementia.

### Step 2: Identification of Relevant Studies

Studies will be retrieved by searching various electronic databases, including PsycINFO, MEDLINE, Web of Science, CINAHL, Scopus, Embase, and Google Scholar. In addition, reference lists of relevant studies will be searched to identify any additional journal articles. The initial keywords that will be included in our search strategy are outlined in Table 1. We will also consult with a research librarian to inform our search strategy and provide expertise to ensure that no relevant databases or search words are missed. More specifically, each database (eg, MEDLINE) may require its own search strategy using Medical Subject Heading terms, which may greatly improve our search results. Our search timeline will focus on peer-reviewed journal articles published from January 1, 2008, to January 1, 2023. This time frame was selected to ensure that our findings are representative of relevant theories and methods that are currently being used to define and measure ageism and stigma of dementia.

**Table 1:** Keyword search strategy. Databases and search engines used were PsycINFO, Web of Science, MEDLINE, CINAHL, Embase, and Google Scholar.

Concept	Keywords
Older adult	Older adult* OR Older adulthood* OR Aged* OR Aging
Dementia	Dementia* OR Cognitive impairment* OR Alzheimer’s disease* OR Parkinson’s disease* OR Cognitive aging*
Stigma OR Ageism	Stigma* OR Ageism* OR Attitude* OR Stereotype* OR Discrimination* OR Bias* OR Prejudice*
Definition	Define* OR Definition*
Measure	Measure* OR Indicator*

Our inclusion criteria will focus on the following four parameters: (1) articles that are written in the English language; (2) full-text, peer-reviewed journal articles; (3) studies that include a definition of ageism and stigma or includes measurements to study the psychometric properties of ageism and stigma; and (4) research that focuses on older adults living with dementia (aged ≥60 years). Given that young onset dementia only occurs in approximately 2%-8% of people before the age of 65 years, we have decided to focus our review specifically on older adults with dementia [22].

### Step 3: Study Selection

We will use Rayyan [23], a leading software tool, to help manage our data and organize our reviews. Following our inclusion criteria, 2 reviewers will independently conduct title and abstract screening. The 2 reviewers will be delegated to oversee the full-text screening of the articles. Any questions on the inclusion of articles will be resolved by discussion with a third reviewer. However, any remaining issues of uncertainty will be resolved through consensus with the full research team. Reasons for study exclusion will be documented and reported in our scoping review publication. The results of our scoping review will be presented using the PRISMA-ScR (Preferred Reporting Items for Systematic Reviews and Meta-Analyses for Scoping Reviews) checklist [24].

### Step 4: Data Extraction

Data will be extracted from the included studies using a systematic extraction approach. More specifically, a standardized data extraction form will be developed and used to chart data from the included studies. Our data extraction form will focus on collecting information on the definitions and measurements for ageism and stigma of dementia as well as identifying any similarities or distinctions between ageism and stigma of dementia (ie, in intersectionality or in assessment measures). The following data may also be included in the form: authors, date of publication, study objective, sample size, sample characteristics (eg, age and gender), study design, and study results. This data extraction form will be pilot-tested with a small number of included studies and revised as necessary. Any modifications or revisions to our data extraction form will be described in our scoping review paper. To reduce errors and ensure consistency in data extraction, one author will oversee the data extraction process. All data collected and analyzed during our scoping review will be made available on an open repository (eg, Open Science Framework) and included as supplementary files with our scoping review manuscript.

### Step 5: Collating, Summarizing, and Reporting the Findings

The analysis will synthesize the evidence based on the existing literature of ageism and stigma toward older adults with dementia. Guided by the work of Arksey and O’Malley [21], our scoping review will not evaluate the methodological quality and rigor of the included studies. Using tables, findings will be presented to showcase the summarized results based on our data extraction findings. Moreover, our findings will highlight research gaps that remain underinvestigated and require further investigation.

## Results

This scoping review was registered with the Open Science Framework on January 17, 2023, prior to any title and abstract screening. Data collection and analysis and manuscript writing is scheduled to occur from March to September 2023. The target date for manuscript submission will be October 2023.

## Discussion

### Principal Results

Addressing issues of intersectional ageism and stigma are critical to improving the quality of life of older adults with dementia. To our knowledge, our research will be the first scoping review to examine and compare the definitional components and measures of ageism and stigma of dementia. However, developing interventions to mitigate intersectional ageism and stigma of dementia requires evidence-informed research of the existing definitions and measures. This scoping review will provide a comprehensive synthesis of the commonalities and differences of the definitions and measures used to address ageism and stigma of dementia. Moreover, this review will shed light on the existing knowledge gaps and the current state of the research, which may help to inform future research, programs, and policy responses.

Our knowledge translation and dissemination strategies will focus on a range of methods, including peer-reviewed journal articles, webinars, and conference presentations. We will also collaborate with our national research networks to disseminate our review’s findings. The reach of our webinars and networks will enable us to develop widespread knowledge dissemination to inform policy, practice, and research.

Although we will aim to conduct a comprehensive scoping review, it will not be without limitations. For example, an important limitation of scoping reviews is that they focus on mapping and synthesizing data rather than evaluating the strength of the evidence or assessing risk of bias in the research. Thus, further research is required to evaluate and assess the quality of the existing studies on ageism and stigma of dementia. Another limitation is that our scoping review will only include peer-reviewed journal articles; as such, it is possible that relevant grey literature may be missed and excluded from our findings. Moreover, articles that are not written in the English language will also be excluded from our scoping review. Consequently, additional research examining grey literature and non-English articles may be useful for developing further insight on intersectional ageism and stigma of dementia.

### Conclusions

This review will synthesize and compare the core definitions and measures used to examine ageism and stigma of dementia. To our knowledge, this study will be the first scoping review to examine ageism and stigma toward older adults living with dementia. Our study will shed light on the existing knowledge gaps and the current state of the research. This knowledge will enable researchers, clinicians, and policy makers to better understand ageism and stigma of dementia. Consequently, the findings from our review may provide pertinent knowledge and insight to inform future research, programs, and policies to address issues of intersectional ageism and stigma of dementia.

## Abbreviations

PRISMA-ScR: Preferred Reporting Items for Systematic Reviews and Meta-Analyses for Scoping Reviews

## Data Availability

All data collected and analyzed during our scoping review will be available on the Open Science Framework repository and included as supplementary files with our scoping review publication.
